# Practical Observations in Surgery and Morbid Anatomy; Illustrated by Cases, with Dissections and Engravings

**Published:** 1817-08

**Authors:** 


### PART II.

### COMPREHENSIVE ANALYTICAL REVIEW
### OF
### MEDICAL LITERATURE.

"Tros, tyriusve, nobis nullo discrimine agetur."

*Practical Observations in Surgery and Morbid Anatomy; illustrated by Cases, with Dissections and Engravings.* By JOHN HOWSHIP, M. R. C. S. &c. &c. &c. *Octavo pp.* 494. *London,* 1817.

WE have seen many persons who had travelled from "Dan to Beersheba"—nay, who had circumvolved the greater part of this globe, without meeting a single incident that could elicit a remark—a single object that could excite a sensation beyond what would have been felt at their own fire-sides at home. So in the medical world, we daily observe men who have been, year after year, in full practice, without encountering a disease that required the exertion of a thought, beyond the *number* of draughts, or *size* of mixtures to be thrown in; without seeing a phænomenon beyond the pale of Cullen's descriptions; or without once dreaming that *Morbid Anatomy* could possibly throw a gleam of light on any tragic scene in the drama of their professional career. To render things still more easy and pleasant, they hit on a technical or artificial pathology that proves extremely useful to them on all occasions: Thus, during the first years of childhood, *teething* is not merely the *cause* of every disease, but it is the disease *itself,* in every instance, excepting small pox, measles, and hooping cough, which cannot easily be placed to the account of dentition. In the ulterior stages of existence, coughs, rheumatisms, indigestion, and *debility* comprise nearly all the ills which flesh is heir to; and the modes of treatment are *varied* in proportion. But there is another class of practitioners, who observe with accuracy—who reason and think on what they see; and who carefully and faithfully record the result of their observations. These are they who ultimately secure the public approbation, and the respect of the profession. It is astonishing what an advantage they possess over their indolent brethren, in every case of difficulty and uncertainty; and what resources are opened to them by the habits of reflection and study, engrafted on observation and experience!

These remarks are called forth by the volume before us, which evinces, in every page, the unwearied industry of its author in watching the phænomena of disease, and recording in the language of truth and simplicity their ever varying forms. Rarely indeed have we seen such an immense body of interesting facts collected into an equal space, as Mr. Howship's work presents; and he who neglects to make himself acquainted with the histories and morbid dissections here recorded, deprives himself of a fund of information which years of personal experience, even on the most extended scale, cannot counterbalance.

Mr. Howship has not only watched the changes of disease at the bed-side of sickness, but has been permitted to avail himself of other sources of information most interesting and valuable—namely, the selection of such cases and appearances of disease as were most to his purpose, from the extensive collection of preparations and their histories, preserved in the invaluable museum of his celebrated friend and patron Mr. Heaviside. The value of this permission has been enhanced by the unrivalled power of Mr. Howship's pencil, in delineating the difficult features of *Morbid Anatomy*, the importance of which is such, that, as Mr. H. justly observes, it may be compared to the Sun, which diffuses an equal and steady light over every path. The Physician or Surgeon, whose steps are not guided by this light, is liable every hour to wander in darkness and error; hence the importance of connecting the phænomena of diseases during life with the *post mortem* appearances.

Mr. Howship has adopted the arrangement of Sandifort in his Museum Anatomicum, which is the order followed in Mr. Heaviside's Museum; namely, according to the natural situation of parts. This work contains the detail of more than one hundred and twenty interesting cases and dissections; it consequently defies regular analysis, and permits us only to offer *samples* of the materials with which so vast a magazine is stored.

Case I. [Case 5 in the work.] *Anchylosis of the Jaws.* The wonderful power of the constitution in compensating for natural defects, or artificial derangements, is here strikingly exemplified. Robert Kilveroy, aged 56 years, had been totally unable to move his jaws from the age of four years! The anchylosis was brought on at that early period by violent inflammation on both sides of the face, followed by exfoliations from about the articulation of the lower jaw. He never experienced a day's sickness for fifty years, although there was no mastication of his food during all that period. In eating, he was in the habit of thrusting in his food with his fingers, by the left side of his mouth, where several of the teeth were deficient. A plate of the anchylosis is presented.

Case II. [8.] *Large Ossific Tumour of the Face.* (Preserved in Mr. Heaviside's Museum.) Eleanor Allway, æt. 30, was admitted into the Westminster Hospital in 1783, with a most extraordinary swelling on the right side of the face, producing great distortion, but no discolouration of the skin. The base of the tumour reached from the eye to the chin; the angle of the mouth depressed, and thrown out of its line; the nose pressed aside. The tumour projected four inches beyond the general line of the facial bones. The affection had extended across the roof of the mouth and boney palate, nearly to the opposite teeth. It was very large and fleshy. The teeth of the upper jaw, thrown out of their natural situation, formed an angle with the remaining part of the alveolar circle; and all those teeth involved in the extent of the tumour, were thus forced into the middle of the mouth, greatly impeding deglutition. This terrible disease had begun about five years before, with a small soft swelling in the right nostril; in which state it produced no uneasiness. On the presumption of its being a polypus, the tumour had been partially extracted at different times; but these operations seemed only to accelerate the progress of the disease, aggravating the degree of uneasiness and pain she now suffered, and hastening the increase of the swelling. When the complaint had become more completely formed, there were two or three teeth which, from their horizontal position, were very much in the way, and troublesome from their being loose. It was considered highly proper that these should be removed; but although this operation required no great effort, it was attended with such an hæmorrhage as brought the patient very low before it could be effectually checked.

*Examination.* On dissection, the tumour proved to be a large fleshy mass or excrescence, surrounding, enclosing, and extending to all the bones attached to the upper jaw, which from pressure suffered a separation at their respective points of union, with such a degree of extension and attenuation of their natural substance, that even the strongest parts of the bones were in many places reduced to the thickness of wafer paper. A beautiful engraving of the cranium and ossific tumour accompanies the description.

CASE III. *Malformation of the Bones of the Face.*

"A lady, who had borne several healthy children, was safely delivered of an infant son; but the child was so shockingly deformed upon the face, that it was considered improper to allow of the mother's seeing him; the infant was therefore immediately taken away, and sent to nurse.

"An only son, and heir to a very large fortune, the unhappy state of this child proved a source of great anxiety and distress to the parents.

"When the infant was about a month old, it was determined to take the opinion of one of the most eminent Surgeons in London, who was accordingly requested to go down into the country, and see the child. On examination, the deformity was found to consist in a double hare-lip, a corresponding division of the palate bones along the middle line in the roof of the mouth, together with a considerable portion of bone continued from the anterior part of the septum of the nose, and projected forward far beyond the line of the alveolar process of the superior maxillary bones. This projection of bone was covered on its superior surface with a small slip of skin, attached to the tip of the nose; which slip would have hung pendulous, but for the projection of the jaw thrusting it upward.

"It was recommended that the child should wait till he was three years old; at which period he was brought up to London. The extremity of the projecting part of the jaw was now considerably broad, and had three teeth growing out from it.

"The first operation consisted in dissecting back the central slip of integument attached to the nose, and then removing the projected part of the jaw with the assistance of a fine saw, so as to allow the central slip of integument to fall into its more natural position, and to bring the external appearance of the face into the state of a double hare-lip merely. The operation succeeded extremely well, and was productive of very little inflammation. The next year the common hare-lip operation was performed, by which one side was united with the central slip; and the following season I assisted at the remaining operation, which succeeded perfectly.

"This young gentleman has now reached the age of nineteen years. He is a very fine youth; and in company it would scarcely be observed that there ever had been any defect in the form of his mouth.

"Not having yet reached his twentieth year, the setting in an artificial palate has been postponed; in consequence of which circumstance, his expression in speaking is still somewhat indistinct." p. 39.

This is a gratifying example of the triumph of art over the aberrations of Nature.

In May, 1814, Mr. Howship performed an operation nearly similar to the above, and with similar success.

After relating several interesting cases of Sanguineous Apoplexy, (none of which we shall quote, in consequence of the large space allotted to this subject in our late commentaries on Morgagni) Mr. Howship concludes, that nine cases in ten of apoplexy are occasioned by effusion of blood into or upon some part of the brain. Effusion of lymph or serous fluid upon the surface of the brain in adults is, he thinks, generally speaking, more apt to connect itself with violent head-ach; or, in its more advanced stages, convulsion. Where extreme severity of pain in the head has preceded an attack of paralysis, the case is more hopeless than where the palsy has come on unaccompanied with that symptom; for where no pain has been felt in the head, or only a temporary sense of giddiness, the probability is, that the paralytic affection may be the result of a mere effusion of blood upon the brain; an accident to which we occasionally find the brain able to accommodate itself, so that with the assistance of proper treatment, the functions of the nervous system are restored, and the patient, more or less, perfectly recovers. When, however, violent pains in the head have been the precursors of the attack, there is great reason to dread the existence of inflammation of the membranes of the brain connecting itself with effusion either of serum or pus, neither of which events, when dependent on an internal cause, have ever yet been proved by subsequent dissection, to be compatible with the recovery of the patient.

Case IV. One of the most extraordinary instances of what the brain will sometimes bear in the way of injury and pressure from effused blood, is said to have occurred in the late Mr. ——, the most famous comedian of his day, who two years before his death had a fit of apoplexy, in consequence of which he partially lost the use of his left side, but in a few months recovered sufficiently to return to the stage, and to command the admiration of the audience to as unlimited an extent as ever. He continued performing until he suffered the second attack, which proved fatal.

"On examination of the head, the seat of the first injury was readily discovered; an apoplectic cyst was found extending the whole length of the right hemisphere of the brain, which measured in breadth nearly two inches in one direction, and one in the other. The coagulum formed by the last attack was comparatively small." p. 66.

*On Pain in the Head.* This affection arises from so many causes, that it is difficult to determine, in many instances, upon what ground, as a disease, it should be taken up. Where general plethora operates as a cause, it will speak for itself; and local plethora will enable the practitioner to decide on the methodus medendi. But not unfrequently the disease hides itself, as it were, within the head, so as to become very puzzling to the physician, while at the same time it requires the greatest promptitude and decision.

Congestion in the head, with its worst effects, extravasation of blood, inflammation, and effusion, is particularly apt to arise from affections of the mind. To explain this fact, we must consider that the brain is the immediate seat of mental perception and feeling; upon which account, it is not at all surprising that it should be most quickly and powerfully subject to the influence of the depressing passions; and as these affections operate by diminishing the energy of the circulation through the whole body, it is natural to expect that the brain should be liable to suffer more immediately and severely, in these complaints, than other parts of the machine; and that this is the case, is a truth, which every day's experience tends to confirm.

CASE V. "Miss C. a single lady, of fair complexion, and tall stature, 35 years of age, always punctual and regular in her menstrual health, had for six or seven years been subject to an uneasy sense of fulness and oppression about the head, sometimes attended with pain and giddiness.

For these complaints, she was in the habit of being occasionally blooded; and for several years, had lost six or eight ounces of blood, every three, four, or six weeks, according to the severity of her head-ach. This treatment always procured temporary relief, but circumstances conspired to favour the continuance of the disorder. About the period of its commencement, she was said to have suffered a disappointment, in a matter which was of the highest concern to her future happiness in life; besides which misfortune, her family were more or less at variance with her, and her subsequent removal from her father's house only served to widen the breach, and increase the frequency of the fits of low spirits, to which she was now obviously falling a prey.

"At one period she was reduced to a state that was completely dropsical, from the frequency with which she insisted upon having blood taken away, to relieve her head. Her limbs became swelled with anasarca. By adopting a change of measures, however, these consequences of extreme debility were removed.

"The necessity for this frequency of bleeding, was considered the more remarkable, because her habits were known to be constantly those of extreme temperance.

"In January, 1813, she had been very low, and had for some days, suffered greatly from the pain in her head. On the Sunday she attended church; but on returning home, said she was very ill, and wished very much to lose some blood. In retiring to her chamber, she told her waiting maid, that she had a severe pain at her heart, and about the shoulders, and that she was persuaded, from the strangeness of her sensations, she was struck with death. She lay down upon the bed, to compose herself; and her attendants were struck with astonishment and terror, on finding, soon afterwards, that she was not asleep, but dead.

"The apothecary in attendance had been with all haste sent for, but came too late. As, however, it appeared proper at least to attempt something, he opened a vein in the arm; but no blood followed.

*Examination.* "Upon opening the head, all that I could observe was an excessive fulness of the vessels in general; both arteries and veins upon the pia mater; and also a certain degree of serous effusion under the tunica arachnoidea, between the convolutions of the brain. The quantity of this serous fluid was altogether, I think, about equal to an ounce.

"The whole of the brain was examined with attention, but the structure appeared to be perfectly sound.

"In the ventricles there was no accumulation of fluid whatever.

"In the thorax the appearances were those of health; nor were there any traces of disease to be found about the viscera of the abdomen." p. 71.

CASE VI. *Convulsion with extreme Debility, treated successfully by Depletion.* Mrs. C. a soldier's wife, ætat. 28, small, delicate; was exposed, in the severe winter of 1808, for several days and nights, to cold and snow, with an infant at the breast. She performed a long journey, and was several times nearly overwhelmed in the snow. Previously to this she had been shipwrecked, and narrowly escaped with life. Having reached Scarborough, where her husband and family resided, her disorder seemed to be merely exhaustion. The pulse was small, low, and slow; the skin cold, and pale; tongue clean and moist; appetite trifling; head-ache; want of sleep. To restore the balance of the circulation, small doses of antimony were exhibited, and repose enjoined; but the latter injunction was not complied with.

*January* 14, 1809. After exhausting exertions, she fell suddenly down, and was found insensible on the floor. She was carried to bed, where she lay tranquil, as if asleep. The face was pale, sunk, and cold, with occasional tremors of the facial muscles,. *Evening.* Still insensible; occasional contractions of the muscles of the limbs, when the pulse became sensibly harder. In half an hour after this, she revived, and complained of excessive darting pains in the head and eyeballs. Could not bear the light; roaring noise in the ears; countenance still sunk; complexion, paleness itself. About three ounces of blood were, with difficulty, abstracted from the arm; a similar quantity was obtained from the other arm. She felt materially relieved. In an hour, she was seized with a rigor and shivering resembling the first stage of ague, which went off in half an hour. Head shaved and covered with a blister. Thirty drops of laudanum procured her some sleep. Next morning she was something better; purged with senna and salts. In the evening, her eyes closed on a sudden; her limbs became rigid; and she was repeatedly convulsed. The convulsion subsiding, she complained again of noise in the head, and extreme sensibility to light. The head-ache is not, as before the bleeding, constant, but transitory and darting. She feels more free and clear in her mind. Saline draughts, with ten drops of tincture of opium in each. 16th. Occasional tendency to delirium; in other respects better. *Cont. medicament.* In the evening, and at precisely the same hour as on the preceding day, she had a return of spasms, during which her eyes were fixed, and the eyelids set wide open; she was pale and insensible; but very soon recovered. A puffy swelling of the face had come on during the day, which was very painful and tender; pediluvium. She continued to improve, and after some trifling relapses, recovered.

Mr. Howship thinks, that the above case furnishes a strong instance of congestion of blood in the head arising principally from mental distress and corporeal sufferings. It would appear, that while the circulation within the head was suffering from oppression, every other part of the vascular system was almost literally emptied of its blood. It is remarkable, that the arteries of the brain evinced no disposition whatever to action, till they were partially emptied by venæsection and other evacuations.

" Then indeed, says our author, there were pains in the head, more decided irritability about the nerves of sense, and other marks of reaction, which, in my opinion, may be much more justly considered an effort of Nature to reassume her proper functions in the œconomy, than as evincing any disposition to disease." p. 98.

Case VII. *Slight Injury of the Head producing Death forty Years afterwards.* (From Mr. Heaviside's Collection.) " In 1792, I was desired to examine the head of Mrs. E———n, who had died the day before, and whom I had attended with Dr. Turton and Dr. Hawey, about eight months before her death, having made her various setons, issues, &c. by their direction. Her case and appearances were the following.

" She was about fifty, the widow of the late Bishop of D---. When about fifteen, being at play, she received a slight tap, rather than a blow, on the right side of her head. It gave her at the moment severe pain; but she disregarded it, and no immediate consequences of any kind followed more than a common headach, commencing always in the part stricken.

" For above thirty years after, she was subject to these attacks, and then began to grow heavy, and sometimes stupid and sleepy, without any known additional cause, though she was naturally one of the liveliest and most witty women existing.

This disposition continued gradually increasing till, for the last year and a half, it was very difficult to keep her awake; but when she was awake, as I have often known, though it was but for half an hour, she had all her natural brilliancy of conversation about her; then all at once would drop asleep again, not to be roused. In this way she went on, till a perpetual comatose state took place and she died convulsed.

" Latterly, her vision had become very much, although very gradually, impaired.

*Examination.* " On examining the head, as soon as I had removed the scalp over the right parietal bone, I saw a portion of the bone, about the size of a crown-piece, seemingly of a very dark colour, directly under the part where the blow had been originally received, and to which spot she invariably pointed as the seat of her pain. On removing the right parietal bone, I found that part of it which appeared discoloured, was transparent, and almost wholly absorbed. It had that colour given it from the portion of the right hemisphere of the brain directly under it being perfectly black, and the colour appearing through the bone, for the dura mater at this part was altogether removed by absorption. Had she lived much longer, I am clear the bone also would have been altogether absorbed, and the brain itself protruded.

" The portion of brain under the seat of the injury was indurated and scirrhous, and this change had taken place through the whole of the middle lobus cerebri. The colour was a dark livid hue.

" Every other part of the cerebrum and cerebellum were perfectly sound, nor was there any disease whatever in the contents of the abdomen or thorax. Nothing but the disease above described, which had so pressed on the optic nerves at their origin as to have made them as flat as a piece of tape, and thereby occasioned her loss of sight, which amounted to almost total darkness for some time before she died.

"How far the tap of the cane, almost 40 years before, brought on this train of symptoms, it may not be easy to decide, and yet it should seem to have had a part in it, by the pain having never varied from the spot where the blow was originally given." p. 121.

We shall continue the Analysis of this very interesting volume in our next Number.

---

